# Portable Spectrometers Give On-Site Drug Testing a Boost

**DOI:** 10.1021/acscentsci.3c00121

**Published:** 2023-02-07

**Authors:** Carolyn Wilke

In February 2022, a police department in Texas announced that it had busted a truck driver for hauling some 2,600 L of liquid methamphetamine. Using tests that mixed the substance in question with tubes of reagents, officers from the Pharr Police Department and the U.S. Drug Enforcement Agency found that the cargo tested positive for the illicit drug. Accused of transporting about $10 million worth of drugs, the driver, Juan Carlos Toscano Guzman, spent almost 6 weeks in jail, the *Fort Worth Star-Telegram* reported. But the truck’s load wasn’t meth.

**Figure:**
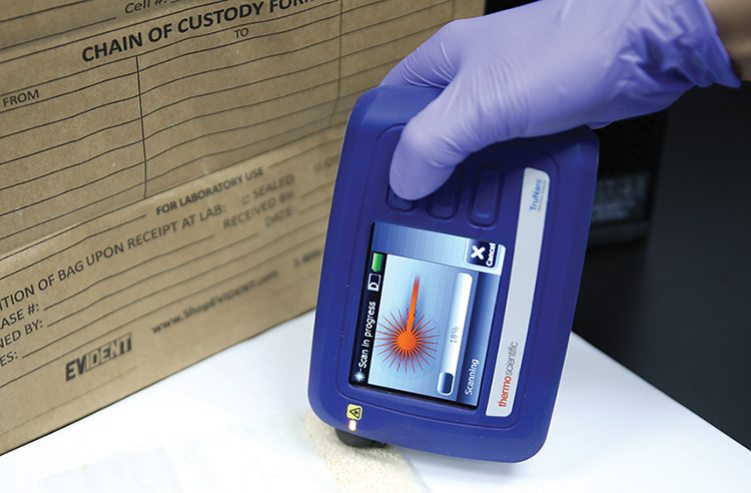
Drug enforcement officers in the field can simply point the TruNarc handheld narcotics analyzer at a sample and get a readout of known drugs that match the sample infrared spectrum. Credit: Thermo Scientific.

Colorimetric test kits had ensnared another individual. Used since the 1970s, the tests are available for dozens of drugs but don’t always provide reliable results. At least 100,000 people across the U.S. plead guilty to possessing drugs after positive field tests each year, according to a ProPublica estimate, so even a modest error rate—due to officers’ lacking proper training, mixing reagents in the wrong order, or getting a false positive—could mean that thousands of people’s lives are unfairly upended.

In establishing the possibility of a drug’s presence, color tests do what they’re supposed to do, says forensic scientist Brooke Kammrath of the University of New Haven. “But they’re misunderstood by the general population and the people who are using them.”

What ended up exonerating Guzman were laboratory tests. He was transporting a mix of oil and diesel, according to his lawyer. Lab methods such as Raman spectroscopy, infrared (IR) spectroscopy, and mass spectrometry are selective and more reliable methods for identifying drugs, but the delay in analyzing samples in the lab can slow investigations and leave innocent people like Guzman behind bars.

Portable versions of such spectrometers are available for police and other drug enforcement agents to use on site, but miniaturizing analytical tools can come with trade-offs in resolution and sensitivity. To make up for that, some chemists, forensic scientists, and even data scientists have started working on ways to extract more—and more accurate—information from field samples collected by police. Some scientists have played a vital role in encouraging the equipment’s availability and adoption and in helping police understand how portable instruments can make law enforcement easier and more fair. “These are sophisticated scientific tools that we’re putting in the hands of potentially nonscientists,” Kammrath says. Scientists need to understand and explain the instruments’ advantages and limitations to ensure the equipment is being used properly, she says.

## Shrinking spectrometers

Portable spectrometers have long been used by nonscientists. Some of the earliest spectrometers that could be taken into the field appeared in the 1950s, including a portable IR spectrometer the U.S. military developed to detect chemical warfare agents. Such instruments became common in the tool kits of U.S. hazmat and threat response teams in the wake of the 2001 anthrax attacks that started a week after 9/11. Today’s array of portable instruments includes optical spectrometers, such as Raman, near- and mid-IR, mass spectrometers, and ion mobility spectrometers.

Especially in the past 20 years, portable instruments have gotten smaller. Some mass spectrometers have shrunk to the size of a briefcase, while some Raman and IR spectrometers can be a bit larger than a deck of playing cards. “The whole revolution in consumer electronics has helped these enormously,” says Richard A. Crocombe, a spectroscopist who runs his own scientific consulting firm. Diode lasers, such as those developed for CD and Blu-ray players and other advances in telecommunications, have helped optical spectrometers slim down. And mass specs have benefited from smaller ion traps. With smaller components that can run on less power, the devices’ footprints have dwindled.

But generally, “portable instruments are not the same as their benchtop counterparts,” Kammrath says. Portable Raman spectrometers can’t yet achieve the throughput and sensitivity that benchtop systems can, and gas chromatography/mass spectrometry (GC/MS) instruments are limited by the type and length of columns available. Resolution can be an issue, for instance, with high-pressure mass spectrometry, in which mixture components aren’t separated before analysis. For comparison, some benchtop mass specs have resolutions eight times as good as these machines.

When it comes to illicit drugs, people who clean up clandestine labs also use such instruments to test whether the unidentified substances they encounter are dangerous. Both IR and Raman spectrometers are simple and fast to use, but IR techniques require the sample to be placed in contact with the detector. For Raman, “you can have a plastic baggie of raw materials, and you can shoot right through it and get a spectrum,” says Pauline E. Leary, a spectroscopist at Noble Supply and Logistics, which sells equipment for military applications and low-resource environments including spectrometers.

At the same time, each portable device has benefits and limitations: GC/MS can parse complex mixtures but destroys the sample, whereas Raman doesn’t. Fluorescence from a sample can mask a drug’s Raman signal, while water in a sample can overwhelm an IR spectrum. So combining multiple tests to create a tool kit is the best approach, Kammrath says. A recent study formed a tool kit containing hand-held or portable Raman, Fourier transform IR (FT-IR), and mass spec devices. Researchers at the U.S. Food and Drug Administration found that, using at least one of the three instruments, they could detect 81 of 88 different active pharmaceutical ingredients. When at least two techniques were used, at least one instrument detected all ingredients. Overall, the tool kit’s results were as reliable as a full-service lab.

**Table 1:** Methods of Portable Chemical Analysis for Drug Testing^a^

portable method	up-front cost	sample handling	data acquisition time	destructive?	target applications	problematic samples
Raman spectroscopy	$12,500–$25,000	Scans through glass and quartz containers and transparent plastics	Few seconds to 1 min	no	Single-component samples, high-concentration mixtures, white powders, liquids, and tablets	Dark, colored, and fluorescent materials, mixtures with low concentration of components (e.g., pills with trace fentanyl), plant samples (e.g., marijuana)
Near-infrared (NIR) spectroscopy	$2,000–$37,500	Scans through glass and quartz containers and transparent plastics	5 s	no	Single-component samples, high-concentration mixtures, white powders	Mixtures with a low concentration of components
Infrared (IR) spectroscopy	$25,000–$50,000	Must be in contact with sample; cannot scan through glass or plastic	< 1 min	no	Single component samples, white powders, liquids, and tablets	Mixtures with a low concentration of components, samples containing water
Ion mobility spectrometry (IMS)	$10,000–$37,500	Takes in a swab of a surface or external packaging	10–30 s	yes	Trace amounts of analytes, high-concentration mixtures; can tolerate mixtures	Samples with concentrated components (e.g., purified powders) that can overload the detector
High-pressure mass spectrometry (HPMS)	>$50,000	Takes in a swab of a surface or external packaging	10–30 s	yes	Trace amounts of analytes; can tolerate mixtures	Samples with concentrated components
Gas chromatography/mass spectrometry (GC/MS)	>$50,000	Takes in sample that is been removed from packaging and dissolved in solvent	4–15 min	yes	Trace amounts of analytes and separation of mixtures	Plant samples that are not dissolved, samples with concentrated components

a Workhorse methods of portable chemical analysis need to be fast and require little sample prep. Each comes with trade-offs in sensitivity and possible application. Source: *Landscape Study of Field Portable Devices for Presumptive Drug Testing*, Forensic Technology Center of Excellence, 2018; Richard Crocombe, The Ever-Shrinking Spectrometer: New Technologies and Applications. In *Sense the Real Change: Proceedings of the 20th International Conference on Near Infrared Spectroscopy*, Xiaoli Chu et al., Eds.; Springer Singapore, 2022), DOI: 10.1007/978-981-19-4884-8_2; *Spectroscopy Outside the Laboratory*, 2022, DOI: 10.56530/spectroscopy.lz8466z5.

## Finding the signal in the noise

Forensic scientists scanning a crime scene may need to see what’s hardly there—trace powders, residues in a container, dopants that make up a small part of a mixture found in the field. The spectra they get from portable instruments often can’t identify a very small amount of a substance among the noise caused by, say, other ingredients in a drug, Leary explains.

U.S. law enforcement agencies have recently seized large amounts of low-dose fentanyl pills. Some of these pills had 1% or less of the synthetic opioid and mostly contained the Tylenol ingredient acetaminophen. According to the U.S. Drug Enforcement Agency, less than 2 mg of fentanyl can be a fatal dose. Instrument manufacturers claimed their equipment could detect fentanyl in such pills, but Leary and Kammrath found that most of the techniques fell short when used in tests.

Acetaminophen and fentanyl have similar IR peaks, and both IR and Raman can’t detect concentrations as low as 1% anyway. With some portable mass spectrometry methods, the acetaminophen would overwhelm the detector. Ion mobility spectrometry could detect 1% fentanyl in the mixture, but the technique isn’t considered the most reliable because unrelated ions of a similar size and weight could have similar mobilities as those of a drug. “A lot of times for these field instruments, we just can’t get the limits of detection we need for a specific problem,” Leary says.

To remedy such problems, Kammrath and her colleagues are trying to come up with new ways to extract trace fentanyl from a mixture so it can be analyzed in the field with a more discriminatory technique. Their working prototype is based on an extraction system from RedWave Technology, a company that develops portable FT-IR instruments. It hinges on a portable tool that takes a powder or pill and does a solvent extraction to concentrate any fentanyl present. An officer could then paint the resulting solution onto the IR detector for a scan. Extraction techniques could potentially expand the range of samples that can be analyzed by portable IR spectroscopy, Kammrath says. Of course, extractions aren’t one size fits all, so new tools would have to be developed to extract other trace drugs.

Parallel to efforts to physically concentrate samples, researchers are also finding ways to unmask components hiding in mixtures by digitally parsing their raw spectral data. There was a time when searching for tricky-to-spot spectroscopic features was like “chasing a ghost,” says Igor K. Lednev, a laser spectroscopist at the University at Albany. For instance, peaks from some components in a mixture could be rendered invisible by the spectral contributions of substances present in much higher concentrations, like in the case of pills with trace fentanyl. “Now, if we combine Raman spectroscopy with statistical analysis, we can reliably detect and identify components in a mixture which you don’t see with the naked eye,” Lednev says.

This approach relies on matching spectral data against databases of known compounds. But sometimes a dangerous drug, such as a fentanyl analog, may be missing. “That particular fentanyl analog may be completely new, and it’s not in our set of what’s familiar,” says Phillip Koshute, a data scientist at the Johns Hopkins Applied Physics Laboratory. He and colleagues have developed machine learning approaches to detect such drugs’ signals lurking in mass spectra and Raman spectra. Working with chemists to zero in on the most important spectral features, the researchers trained machine learning models on pure substances’ spectra to detect fentanyl analogs. “The next step would be repeating the process but with the real-world, messy data,” Koshute says.

The capability to analyze mixtures or identify novel compounds could someday be built into the instruments, Lednev says. Portable spectrometers are already equipped to transmit their data wirelessly. Spectra could be sent to a cloud-based tool for machine learning, returning a determination and confidence interval.

## In the field

Compared with hazmat teams and fire departments, “the forensic community has been very slow to adopt portable instruments,” Kammrath says. But some police departments and crime labs are starting to take to the devices.

Since 2011, dozens of agencies around Alabama have begun using portable Raman spectroscopy for drug testing in the field or the lab. Mark Hopwood was then the director of one of the state’s crime labs, and a backlog of some 30,000 drug cases statewide required testing. “It was taking anywhere from a year to 2 years to get lab results back,” says Hopwood, who is now a forensic scientist at Jacksonville State University (JSU).

In an effort to reduce the backlog, Hopwood’s team tried out portable spectrometers, playing with the devices for a month and doing field trials. Of the systems tested, Thermo Scientific’s TruNarc hand-held analyzer, a Raman spectrometer with only three buttons that looks like a chunky hand-held gaming console, stood out for its ease of use and durability, Hopwood says. Additionally, “there was no way to manipulate data,” he says. For instance, it wouldn’t be possible for an officer acting in bad faith to scan sugar or salt and falsely name it as cocaine or another drug in the spectral library. If a scanned substance came up as an unknown, the team could use what’s called a reachback service, getting support within hours or a day from Thermo scientists, who could help identify the compound and add the substance to the spectral library. Such services are already commonly used by hazmat teams that want a trained analyst to verify results or talk through data concerns, Noble’s Leary says.

The spectra of the scanned substance and its library match could be shared with defense attorneys, who could then advise defendants to either go to drug court or take a plea deal, Hopwood says. A plea deal based on a spectrometry result may be preferable to one based on less-reliable color tests. And in a case where a conviction is likely after lab-based testing, going to court may drag out the legal process, he adds.

The TruNarc devices helped cut Alabama’s pending caseload by some 30% within a few months. “It ended up freeing up the jails, saving the sheriffs money—because they’re not having to feed and house people,” and the courts were able to collect fines, Hopwood says.

A 2014 survey of portable Raman for drug testing calls Hopwood a “technology champion” for the instruments. He’s helped departments adopt these devices and is training drug task force and narcotics units how to use them. He has also opened his department at JSU to officers from nearby counties, making a few devices available for their use when they need a quick identification.

Funding can hinder police departments in adopting portable spectrometry, Kammrath says. A bill was introduced in the U.S. Congress in 2019 that would have funded departments looking to buy portable instruments for drug testing, but it didn’t garner enough support to move forward.

Kammrath says scientists could help strengthen the argument for these devices and increase their appeal to lawmakers and police. A cost-benefit analysis targeted at law enforcement that details other tangible benefits, such as cost savings from not purchasing color tests, and intangible ones, such as the cost of life from wrongful arrests and incarcerations, could help change minds. “Portable instruments are rapid, they’re reliable, and they create a reviewable record,” she says. “We haven’t made a good-enough case as a scientific community for our need for these instruments.”

## Carolyn Wilke is a freelance contributor to

Chemical & Engineering News, *the independent news outlet of the American Chemical Society.*

